# Awareness of Radiation Hazards and Knowledge About Radiation Protection Among Medical Students at the Northern Border University, Arar

**DOI:** 10.7759/cureus.55484

**Published:** 2024-03-04

**Authors:** Pakeeza Shafiq, Yasir Mehmood

**Affiliations:** 1 Department of Surgery, Northern Border University, Arar, SAU

**Keywords:** arar, medical students, knowledge, awareness, radiation protection, radiation hazards

## Abstract

Studies have shown that medical students and doctors are not well equipped with knowledge of radiation hazards and their protection. This lack of knowledge may cause harm to patients and healthcare professionals.

Objectives

To compare the awareness of radiation hazards and knowledge about radiation protection among medical students at Northern Border University, Arar, Saudi Arabia, with and without prior teaching.

Methods and materials

This cross-sectional study was carried out among medical students from clinical years at Northern Border University, Arar, Saudi Arabia, from May 1st, 2023, to June 30th, 2023. Their consents were taken. Then they were randomly sorted into two groups. One group was given a radiation safety short course, and then they answered a questionnaire. The other group filled out the questionnaire without prior instruction, and analysis was done. The outcome of this study was quantified based on the score calculated after participants filled out the questionnaire.

Results

The mean score of students who didn’t attend the radiology course was 3.38±1.8, while the score of students who attended the radiology course was 7.87±1.4 (p<0.001). Radiology course attendance showed a significant association with knowledge and awareness scores.

Conclusions

The average knowledge of medical students regarding radiation protection and hazards is quite poor. This lack of understanding could potentially lead to increased risks for both patients and healthcare professionals. The knowledge about radiation hazards and protection is increased in students who attended a short radiology course. We recommend implementing comprehensive educational programs that focus on radiation hazards and protection for medical students.

## Introduction

Radiation is the energy that is released or transmitted as waves or particles that can penetrate substances and human beings. Radiation is of two types: ionizing and non-ionizing [1]. High-energy ionizing waves can alter molecules in biological tissues causing damage to genetic and DNA material [2]. Computed tomography (CT), which uses X-rays, is currently the most widely employed method using ionizing radiation in medical diagnosis. More detailed information is provided by CT scans than by standard X-ray examinations [3]. The use of CT scans has increased to 70 million scans per year since 1993 in the US [4].

However, while these radiologic tests are helpful in the diagnosis and treatment of patients, there are certain drawbacks as well. Exposure to high-dose radiation from these machines can also cause radiation dermatitis, hair loss, and ulceration. Sometimes it may result in deleterious damage, such as cancer [5].

Background natural radiation accounts for ∼2.4 mSv/year [6]. The unit sievert (Sv) is used to quantify the amount of damage radiation does to the human body. The unit gray (Gy), on the other hand, is the energy absorbed in the body per unit mass [7]. As future professionals who need to deal with such hazardous environments, medical students should not only have background knowledge about radiation risks but should also know how to deal with them.

The effects of ionizing radiation exposure can be divided into two types: stochastic and deterministic. Stochastic effects do not have a dose threshold and can have outcomes at any dose. The harmful effects can appear after 10-20 years. The severity of the deterministic effect increases as the dose increases. Hence, deterministic effects have a dose threshold. Several harmful outcomes occur depending on the radiation dose when the radiation dose is exceeded [8]. The lifetime risk of cancer increases to one every 100 after exposure to radiation of 100 mSv. Accidental exposure to high-dose radiation can also cause radiation-induced dermatitis, hair loss, and ulceration [9].

Radiation protection includes the protection of people and their environment from the damaging effects of ionizing radiation. Radiation protection works by three rules: distance, exposure duration, and shielding. As you get closer to a radiation source, the radiation's dose rate (intensity) rises. Time and radiation exposure are related. There should be a shielding substance positioned between the subject and the source [10]. The instruments and ways of protection from radiation should be known to everyone. These include area radiation monitors, survey meters, lead shields, and personnel dosimeters for staff [11].

The previous studies conducted on this topic show insufficient knowledge among clinical-year medical students. A study from the UK showed that medical students had poor knowledge about radiation hazards and their protection. Furthermore, the knowledge did not improve with increasing seniority [12]. In a study conducted on 518 medical students in Saudi Arabia, about 60% of the participants did not know the radiation dose of a common imaging modality like a chest X-ray. Almost half of the students (49.6%) have no idea that a radiological investigation, for example, an abdominal computed tomography (CT) scan, when done multiple times, can be associated with a risk of fatal cancer [13]. Considering the lack of awareness mentioned in the previous studies, this study was planned to compare the awareness of radiation hazards and knowledge about radiation protection among medical students at Northern Border University, Arar, Saudi Arabia, with and without prior teaching.

## Materials and methods

This cross-sectional study was carried out among the medical students at Northern Border University, Arar, Saudi Arabia, from May 1st, 2023, to June 30th, 2023. Two hundred and forty-eight medical students of fourth, fifth, and sixth years who consented were included in the study. These students had no prior teaching on radiation hazards and protection. The students who didn’t give consent or incompletely filled out the questionnaire were excluded. They were randomly sorted into two groups. One group was given a radiation safety short course in the form of a lecture, whereas the other group was not offered any such teaching course. A questionnaire was then used to assess the awareness of radiation hazards and knowledge about radiation protection among medical students in both groups (Appendix 1).

The questionnaire was used to gather information in three sections, each serving a specific purpose. The first section focused on demographic characteristics, while the second section addressed radiation hazards. Lastly, the third section was related to radiation protection measures [14]. The questionnaire was converted to an online survey tool. The link was distributed among students in both groups, and responses were collected. The data was entered and analyzed using IBM SPSS Statistics for Windows, Version 20 (released 2011; IBM Corp., Armonk, New York, United States).

Data analysis

All data was analyzed with IBM SPSS Statistics for Windows, Version 20 (released 2011; IBM Corp., Armonk, New York, United States). Qualitative variables were presented in the form of frequency and percentages, while quantitative variables were presented in the form of mean and standard deviation. The Mann-Whitney U test was applied. A p-value of ≤0.05 was considered statistically significant.

## Results

In this study, the mean age of study participants was 21.93±0.76 years. The minimum and maximum ages were 21 and 23 years, respectively. Among participants, 120 (48.4%) were males and 128 (51.6%) were females. There were 87 (35.1%) participants in the 4th year, 98 (39.5%) in the 5th year, and 63 (25.4%) in the 6th year, respectively (Table 1). Knowledge and awareness scores were compared between different characteristics of study participants (Table 2). However, no statistically significant difference was seen in knowledge and awareness scores concerning age, gender, and year of study. Participants who had attended the radiation safety short course had significantly higher knowledge and awareness scores as compared to the participants who had not attended it. The knowledge and awareness score percentage showed no significant association with the age, gender, or year of study of the participants. However, radiation safety short-course attendance showed a significant association with knowledge and awareness score percentage. Participants who had attended the course had better knowledge and awareness score percentages as compared to those who did not attend it (Table 3).

**Table 1:** Study participant's characteristics (n=248)

	Frequency	Percent
Age (Years)	21.93±0.76	
	Min=21, Max=23	
Gender		
Male	120	48.4%
Female	128	51.6%
Year of study		
4^th^	87	35.1%
5^th^	98	39.5%
6^th^	63	25.4%
Knowledge & awareness mean score	5.62±2.78	
	Min=2, Max=10	

**Table 2:** Knowledge and awareness score about study participants’ characteristics (n=248) a: Kruskal Wallis H test; b: Mann Whitney U Test (*): p-value <0.05 (statistically significant)

	Mean ± SD	p-value	
Age			
21 years	5.74±2.80		0.814^(a)^
22 years	5.57±2.77		
23 years	5.57±2.82		
Gender			
Male	5.77±2.78		0.493^(b)^
Female	5.48±2.78		
Year of study			
4^th^	5.76±2.83		0.612^(a)^
5^th^	5.47±2.76		
6^th^	5.68±2.79		
Radiology module attended			
Not attended	3.38±1.81		<0.001*^(b)^
Attended	7.87±1.45		
	Mean ± SD	p-value	
Age			
21 years	5.74±2.80		0.814^(a)^
22 years	5.57±2.77		
23 years	5.57±2.82		
Gender			
Male	5.77±2.78		0.493^(b)^
Female	5.48±2.78		
Year of study			
4^th^	5.76±2.83		0.612^(a)^
5^th^	5.47±2.76		
6^th^	5.68±2.79		
Radiology module attended			
Not attended	3.38±1.81		<0.001*^(b)^
Attended	7.87±1.45		

**Table 3:** Knowledge and awareness score percentage in relation to study participants’ characteristics (n=248) Note: (a) Chi-Square test, p-value<0.05 (statistically significant)

		Knowledge & awareness score percentage								p-value(a)
		<20%		21-40%		41-60%		<70%		
		n	%	n	%	n	%	n	%	
Age	21 years	28	31.10%	14	32.60%	34	32.40%	5	50.00%	0.871
	22 years	37	41.10%	19	44.20%	44	41.90%	2	20.00%	
	23 years	25	27.80%	10	23.30%	27	25.70%	3	30.00%	
Gender	Male	40	44.40%	22	51.20%	51	48.60%	7	70.00%	0.464
	Female	50	55.60%	21	48.80%	54	51.40%	3	30.00%	
Year of study	4th Year	30	33.30%	15	34.90%	37	35.20%	5	50.00%	0.923
	5th Year	37	41.10%	18	41.90%	41	39.00%	2	20.00%	
	6th Year	23	25.60%	10	23.30%	27	25.70%	3	30.00%	
Radiology module	Not attended	88	97.80%	24	55.80%	12	11.40%	0	0.00%	<0.001*
	Attended	2	2.20%	19	44.20%	93	88.60%	10	100.00%

## Discussion

Our study aimed to assess the knowledge of medical students at Northern Border University, Arar, Saudi Arabia, about radiation hazards and their protection. Our study showed that the mean score among students who were not given radiology teaching was 3.38±1.81, while the mean score among students who attended the radiation safety short course was 7.87±1.45 (p<0.001). These results favor our hypothesis that the radiology course improves medical students’ knowledge about radiation protection and risks. There is increasing concern that medical students are generally unaware of these risks and, hence, fail to properly inform patients of the risks and benefits of a given investigation [15]. A Norwegian study showed that the medical students were very poor in their knowledge of radiation protection and its hazards. The total mean score was only 3.91 out of a possible 11. More than half of the students were confident about their knowledge and awareness of radiation doses [16].

The knowledge of fourth- and fifth-year medical students is limited (42.8% and 44.8%, respectively) as compared to house officers and postgraduate trainees (83% and 78.2%, respectively), according to a study conducted in Pakistan [17]. Moreover, based on a study conducted in Saudi Arabia, only 38.7% of the students had enough knowledge about ionizing radiation (IR)-related risks. The majority (76%) had never attended refresher courses on radiation protection [18]. Another study from South Africa suggested including a rotation of the department of radiology to improve awareness of the radiation risks observed in the study [19].

Our study not only intended to assess the level of knowledge among medical students but also worked out a simple solution that can help improve the student’s knowledge about radiation hazards and protection in the future. The knowledge score was 3 in students who did not attend the course, while it was 8 (p<0.001) in students who attended a short radiology course. A study done in Ireland shows that the students who received radiology instructions had a significantly higher number of correct answers as compared to those who didn’t (59.7% versus 38%, p < 0.001) [20]. A previous study done in Saudi Arabia also showed that fourth-year medical students had inadequate knowledge regarding ionizing radiation and radiation protection. They required additional lectures in radiation protection to improve their knowledge about the topic [21]. We need to make efficient amendments to enhance the knowledge and understanding of radiation hazards and protection among medical students, ultimately contributing to a safer and more informed healthcare environment.

This study has provided valuable information, but it has a few limitations. The study has a small sample size, which could affect the generalizability of the findings to a larger population of medical students. It has focused on a specific demographic group of medical students, which could limit the applicability of the findings to a more diverse population. The study has relied on self-reported data from the participants, which can be subject to bias and may not accurately reflect their actual knowledge and awareness of radiation hazards and protection.

## Conclusions

The research findings indicate that the average knowledge of medical students regarding awareness of radiation hazards and radiation protection is poor. This lack of understanding could potentially lead to increased risks for both patients and healthcare professionals. It is imperative to address this issue to improve overall safety and quality of care within the medical field.

The study demonstrated that the students who attended the course had a greater knowledge of radiation hazards and their protection compared to those who did not attend the course. We recommend implementing comprehensive educational programs that focus on radiation hazards and protection for medical students. These programs should be integrated into the existing curriculum and provide practical, hands-on training. This will enhance the knowledge and understanding of radiation hazards and protection among medical students, eventually contributing to a safer and more informed healthcare environment.

## Appendices

Appendix 1

Questionnaire: Awareness of radiation hazards and knowledge about radiation protection among medical students at Northern Border University, Arar.

1.  Age: ___________________

2. Gender: Male / Female                        

3. Year of study: 4            5            6         

4. Nationality:         

o  Saudi

o  Non-Saudi

5. Radiology course: Attended/Not attended

Radiation risk awareness

6. Which of the following investigations is safe during pregnancy?

o  CT

o  MRI

o  Ultrasound

o  X-ray

o  I do not know

7.  Which investigations can be done safely in children?

o  CT

o  MRI

o  Ultrasound

o  X-ray

o  I do not know

8. What is the radiation dose during IVU?

o  1.4 mSv

o  7 mSv

o  10 mSv

o  I do not know

9. Which radiologic tests can give some amount of radiation?

o  CT

o  Fluoroscopic examinations

o  Nuclear examination tests

o  I do not know

10. Which of the following investigations propagate maximum ionizing radiations?

o  CT

o  MRI

o  Fluoroscopy

o  X-ray

o  I do not know

11. What are occupational hazards associated with medical imaging?

o  Cataract

o  Cancers

o  Skin rashes 

o  I do not know

12. Chest X-ray when compared to natural background radiation is equal to

o  10 days

o  2 months

o  1 year

o  3 years

o  I do not know

13. Pregnant women should avoid all types of medical imaging.

o  True

o  False

o  I do not know

14. Who is more at risk of radiation-related sickness?

o  Pregnant women

o  Women

o  Children

o  All

o  I do not know

Radiation protection

15. Which of the following substances are used to coat the walls of a CT scan room for radiation shielding?

o  Tungsten

o  Glass

o  Lead

o  Iron

o  I do not know

16. Which of the following substances are used to make protective shields for radiology workers?

o  Tungsten

o  Glass

o  Lead

o  Iron

o  I do not know

17. What is the effective dose limit of radiation for occupationally exposed workers?

o  20 mSv

o  50 mSv

o  I do not know

o  Other _________

18. Which method is used to reduce the amount of exposure to ionizing radiation?

o  Time

o  Distance

o  Source

o  None of the above

o  I do not know

19. What are the principles for reducing radiation risk?

o  Justification

o  Limitation

o  Optimization

o   All of the above

o   I do not know

## References

[1] Tuieng RJ, Cartmell SH, Kirwan CC, Sherratt MJ (2021). The effects of ionising and non-ionising electromagnetic radiation on extracellular matrix proteins. Cells.

[2] Ünal ES, Geliş K, Baykan P (2018). Investigation of awareness levels about the radiation safety of personnel working in the imaging units of the hospitals in Agri, Turkey. Journal of Radiation Research and Applied Sciences.

[3] Shi L, Tashiro S (2018). Estimation of the effects of medical diagnostic radiation exposure based on DNA damage. J Radiat Res.

[4] Awadghanem A, Sbaih M, Hasoon M, Yassin Z, Samara AM, Maree M, Zyoud SH (2020). An assessment of medical students' proficiency regarding the hazards of radiological examinations on the health of workers and patients: a cross-sectional study from Palestine. J Occup Med Toxicol.

[5] Alreshidi MN, Alshubrmi D, Alreshidi F, Soliman K, Alrashidi I (2020). Knowledge about imaging modalities, risks, and protection in radiology among medical students at the University of Hail. Avicenna J Med.

[6] Ribeiro A, Husson O, Drey N, Murray I, May K, Thurston J, Oyen W (2020). Ionising radiation exposure from medical imaging - a review of patient's (un) awareness. Radiography (Lond).

[7] Schultz CH, Fairley R, Murphy LS, Doss M (2020). The risk of cancer from CT scans and other sources of low-dose radiation: a critical appraisal of methodologic quality. Prehosp Disaster Med.

[8] Zervides C, Sassis L, Kefala-Karli P, Christou V, Derlagen A, Papapetrou P, Heraclides A (2020). Assessing radiation protection knowledge in diagnostic radiography in the Republic of Cyprus. A questionnaire survey. Radiography (Lond).

[9] Hazem M, Al-Omran QA, Al Murawhan BQ, Al Omran AA, Alluwaim MA, Al Saeed AA (2020). Perception of radiation hazards by medical interns in eastern Providence, Saudi Arabia. Inte Jour of Phar and Phyto Res.

[10] Kim JH (2018). Three principles for radiation safety: time, distance, and shielding. Korean J Pain.

[11] Eze CU, Abonyi LC, Njoku J, Irurhe NK, Olowu O (2013). Assessment of radiation protection practices among radiographers in Lagos, Nigeria. Niger Med J.

[12] Khan MO, Khan MS, Janjua O, Ali A, Hussain S (2019). Knowledge of radiation legislation and radiation exposure in common radiological investigations among final year medical students, foundation doctors, specialist radiology registrars and radiographers at a UK university teaching hospital. BJR Open.

[13] Alturki ST, Albusair MK, Aljalajel KM, Alshahrani AS, Albadrani MS, Alhuwaymil AA, Almotairy AS (2020). Awareness and knowledge of radiation in common radiological investigation and associated risks among medical students in Saudi Arabia: a cross-sectional study. Imam J Appl Sci.

[14] Faggioni L, Paolicchi F, Bastiani L, Guido D, Caramella D (2017). Awareness of radiation protection and dose levels of imaging procedures among medical students, radiography students, and radiology residents at an academic hospital: results of a comprehensive survey. Eur J Radiol.

[15] Scali E, Mayo J, Nicolaou S, Kozoriz M, Chang S (2017). Senior medical students’ awareness of radiation risks from common diagnostic imaging examinations. Can Med Educ J.

[16] Kada S (2017). Awareness and knowledge of radiation dose and associated risks among final year medical students in Norway. Insights Imaging.

[17] Maqsood B, Masood HA, Anjum N (2021). Radiation risks and protections associated with imaging modalities. PJMHS.

[18] Alduraibi SK, Alahmad A, Alghadhiyah D, Alduraibi A, Algazlan M, Alduraibi A (2021). Knowledge of radiation safety among medical interns in Saudi Arabia. Inte Jour of Medi in Devel Coun.

[19] Dauda AM, Ozoh JO, Towobola OA (2019). Medical doctors' awareness of radiation exposure in diagnostic radiology investigations in a South African academic institution. SA J Radiol.

[20] O’Sullivan J, O’Connor OJ, O’Regan K, Clarke B, Burgoyne LN, Ryan MF, Maher MM (2010). An assessment of medical students’ awareness of radiation exposures associated with diagnostic imaging investigations. Insights into imaging.

[21] Hagi SK, Khafaji MA (2011). Medical students' knowledge of ionizing radiation and radiation protection. Saudi Med J.

